# The impact of endoscopy on the treatment of cholesteatomas

**DOI:** 10.5935/1808-8694.20130090

**Published:** 2015-10-08

**Authors:** Thiago de Oliveira Lima, Taís Figueiredo de Araújo, Letícia Clemente Alvim Soares, José Ricardo Gurgel Testa

**Affiliations:** a MD. Otorhinolaryngologist. Fellow in Otology - Department of Otorhinolaryngology and Head and Neck Surgery - Medical School of the Federal University of São Paulo - UNIFESP-EPM.; b MD. Otorhinolaryngologists. ENT Residency from the Santa Marcelina Hospital.; c MD. MSc in Science - UNIFESP. Preceptor in ENT - Santa Marcelina Hospital/Otorhinus Clinic.; d PhD in Science - UNIFESP; Adjunct Professor at the Department of Otorhinolaryngology and Head and Neck Surgery - UNIFESP-EPM. Medical School of the Federal University of São Paulo - UNIFESP/EPM.

**Keywords:** cholesteatoma, cholesteatoma, middle ear, endoscopy

## Abstract

Recurrent cholesteatoma is relatively uncommon. Residual middle ear cholesteatomas account for most of the cases of recurrent disease. The limited role of microscopy in the visualization of tridimensional anatomic alterations of the temporal bone led to the use of endoscopic examination as an additional tool in the realm of ear surgery. Endoscopy has significantly aided in the management of chronic cholesteatomatous otitis media and in the prevention of recurrent disease.

**Objective:**

To review the literature and assess the relevance of endoscopy in the surgical treatment of cholesteatomas and in the prevention of relapsing lesions.

**Method:**

Searches on databases MedLine and LILACS were carried out between March and June of 2011 to select studies in which endoscopy was used in the management of cholesteatomas.

**Results:**

Three studies comparing surgery aided by endoscopy and surgery performed with the aid of a microscope met the inclusion criteria.

**Conclusion:**

Endoscopy has positively impacted the management of cholesteatomas and should be used in cholesteatoma surgery.

## INTRODUCTION

Schuknecht^1^ described cholesteatomas as collections of foliated keratin in the middle ear or any aerated area of the temporal bone originated from keratinized epithelium.

In 1874, Jean Petit described the first successful surgical intervention on the mastoid. Since then, the management of chronic cholesteatomatous otitis media (CCOM) has been imminently surgical^2^.

Surgery is required to tackle the continuous growth of cholesteatomas, associated bone resorption, involvement of sensory organs, and potentially fatal intra and extracranial complications.

In the early 1950s, Wüllstein^3^ and Zöllner^4^ set the basic principles of tympanoplasty and established three basic objectives in chronic ear surgery:

• Eradicate disease;
• Preserve or restore auditory mechanics;
• Maintain, whenever possible, the anatomy of the temporal bone.

Various approaches based on these concepts were developed, such as tympanoplasty with canal wall up mastoidectomy and variants of canal wall down mastoidectomy^5^, ^6^, ^7^.

The approaches to cholesteatomatous middle ear include basically tympanoplasty, canal wall up (CWU) and canal wall down (CWD) mastoidectomy^7^, ^8^.

The choice of surgery to treat CCOM is based on complex criteria and a number of factors such as patient age, extent of involvement, middle ear mucosa status, anatomic peculiarities, auditory acuity, and social factors affecting patient follow-up^7^, ^8^.

The most significant concern in the postoperative follow-up of patients submitted to any of the procedures mentioned above is recurrence of cholesteatoma and onset of potential associated complications.

Relapse may occur in cases of residual cholesteatoma or recurrent cholesteatoma.

Residual cholesteatoma has been defined as relapsing disease originated from remnants of cholesteatoma left on the site of surgery^9^. Prevention of residual cholesteatoma requires meticulous removal of the entire disease in the first procedure^5^.

According to Sheehy et al.^10^, the term “recurrent” applies to lesions stemming from epithelial migration secondary to graft continuity defect and originated from tympanic membrane retraction pouches.

The limited role of microscopy in the visualization of tridimensional anatomic alterations of the temporal bone led to the use of endoscopic examination as an additional tool in the realm of ear surgery. Endoscopy has significantly aided in the management of chronic cholesteatomatous otitis media and in the prevention of recurrent disease.

This study aimed to assess, through a systematic review, the importance of endoscopic examination of the ear in the prevention of recurring disease and in the treatment of chronic cholesteatomatous otitis media.

## METHOD

Literature searches were carried out between March and June of 2011 on databases MedLine (National Library of Medicine) and LILACS (Literature on Health Sciences from Latin America and the Caribbean). Priority was given to studies on the application and impact of ear endoscopy as an ancillary method in the management of CCOM. The Cochrane Reviews Handbook^11^ was used to pick the search strategy adopted in this study. Search keywords can be seen on Tables 1 and 2. The search was limited to papers published between January of 1985 and June of 2011.

**Table 1:** Keywords and used combinations.

#1 cholesteatoma	#5 (#1 and #3)
#2 residual cholesteatoma	#6 (#2 and #3)
#3 *endoscopy

**Table 2:** Keywords in Portuguese and used combinations

#1 colesteatoma	#5 (#1 and #3)
#2 colesteatoma residual	#6 (#2 and #3)
#3 *endoscopia	

This study included original papers from studies done on adult (age > 19 years) or pediatric populations, meta-analyses, systematic reviews, comparative or experimental cohort studies published between January of 1985 and June of 2011 (written in English, French, or Portuguese) on the aspects related to the epidemiology, diagnosis, and treatment of residual cholesteatoma associated with endoscopic examination of the ear. Case reports, letters to the editor, papers published in meeting proceedings, and papers in which the analyzed sample was not statistically significant were excluded. The selected original papers were analyzed for compliance with the criteria described above. The references of the selected papers were used to locate papers not found in the initial search.

Data on authors, clinical diagnosis, participants (number of enrolled subjects and group allocations), participant age range, pattern of CCOM, details pertaining to the chosen surgical approach, statistical analysis used to assess impact, outcomes reported during surgery, and pattern of recurrence of the studied disease were summarized for the selected papers.

## RESULTS

The initial database search listed 140 references, 106 of which resulting from the combination of keywords “cholesteatoma” and “endoscopy”; 25 papers were listed from the combination of terms “residual cholesteatoma” and “endoscopy”. Twenty-three papers appeared twice in the cross-referencing of all searches; 108 papers were left after the duplicates were removed. Thirty-seven of these papers were pre-selected based on the contents of their titles and the established inclusion and exclusion criteria. The abstracts of these papers were read and 27 were found to be case reports, non-comparative series reports, papers that could not be located, or papers written in languages other than English, Portuguese or French.

Seven of the remaining papers selected based on the inclusion criteria and method were excluded for describing case reports (Table 3). Three papers looked into the use of endoscopy in cholesteatoma surgery against traditional surgery with the aid of a microscope (Table 4).

**Table 3:** Reason for paper exclusion.

1993, Yanagihara et al.^5^	Case series report; non-comparative study;

2010, Yang et al.^12^	Case series report; non-comparative study;

1996, Gyo et al.^13^	Case series report; non-comparative study;

1999, Tarabichi et al.^14^	Case series report; non-comparative study;

1997, Tarabichi et al.^15^	Case series report; non-comparative study;

2002, Bad-El-Dine^16^	Case series report; non-comparative study;

2008, Barakate et al.^17^	Case series report; non-comparative study.

**Table 4:** Comparative summary of relevant papers.

Author	Thomassin et al.^9^	El Meselaty et al.^18^	Ayache et al.^19^
Diagnosis	Primary/secondary acquired cholesteatoma.	Primary/secondary acquired cholesteatoma.	Primary/secondary acquired cholesteatoma.

Participants	80 (2 groups - 44 submitted to microscope-aided surgery/36 to endoscope-aided surgery).	82 (4 groups - 23 offered CWU mastoidectomy/21 endoscope-aided CWD mastoidectomy/21 CWD mastoidectomy/21 endoscope-aided CWU mastoidectomy).	247 (167 submitted to microscope-aided surgery/80 to endoscope-aided surgery).

Age	60 adults/20 children.	6-58 years.	3-87 years.

Notes on the study	Patients were randomly assigned to groups regardless of extent of disease or type of surgery.	Patients were randomly distributed in groups based on type of surgery.	This retrospective study included 247 individuals with epitympanum involvement. Patients were randomly assigned to groups regardless of type of surgery.

Program details	All subjects underwent endoscope-aided minimally invasive surgery between 13 and 19 months after the initial procedure.	Mean follow-up of 19.20 (± 8.7) months; only patients followed up for at least a year were included.	40% of the subjects in group I and 45% of the individuals in group II were reoperated one year after the first procedure; they were picked randomly. The other subjects were followed up conservatively.

Findings	Recurrent cholesteatoma in 47% of the cases vs. 6% among members of the group submitted to endoscope-aided procedures.	No cases detected in patients operated with the aid of an endoscope vs. 5-26% recurrent disease in patients operated with the aid of a microscope.	No significant differences between groups; importance of good disease removal in endoscope-aided procedure was stressed.

Thomassin et al.^9^ studied 80 patients with acquired cholesteatoma randomly divided regardless of extent of involvement or type of planned intervention, as follows:

• Group I (n = 44), submitted to microscope-aided CWD or CWU mastoidectomy;
• Group II (n = 36), submitted to similar procedures as described above with the aid of endoscopic examination of the ear to detect residual cholesteatoma on the site of surgery.

In order to verify cases of relapsing disease, the subjects in both groups underwent endoscopic minimally invasive procedures between 13 and 19 months after the original procedure. Significant differences were seen between groups. Forty-seven percent of the individuals in group I had recurrent cholesteatomas, against six percent of the subjects in group II. The comparison also revealed that subjects in group II had minor residual cholesteatomas - small or pearl-shaped cholesteatomas - whereas 10% of the individuals in group I required additional surgery due to the size of their relapsing lesions.

El Meselaty et al.^18^ studied 82 patients aged between six and 58 years with acquired cholesteatoma. The subjects were divided into four groups according to the procedures they were offered.

• Group I (n = 23) subjects were submitted to traditional CWD mastoidectomy without verification of residual disease during surgery;
• Group II (n = 21) subjects underwent CWD mastoidectomy with endoscopic intraoperative verification and removal, if needed, of residual disease;
• Group III (n = 21) individuals were offered traditional CWU mastoidectomy;
• Group IV (n = 21) individuals were submitted to CWD mastoidectomy with the aid of endoscopic examination.

The analysis of recurrence rates was based on the data gathered during a mean follow-up of 19.20 (± 8.7) months. The author included in his study only the patients who had been followed up for at least a year. Subjects in group I has recurrence rates of five percent, against 26% of individuals in group III; no recurrence was observed in the patients operated with the aid of an endoscope.

Ayache et al.^19^ published a retrospective study including 350 patients aged between three and 87 years. The subjects underwent tympanoplasty, transmeatal approach mastoid surgery, CWD or CWD mastoidectomy depending on the extent and location of disease. Involvement of the epitympanum was observed in 247 individuals. They were randomly assigned to two groups, namely groups A (n = 167) - microscope-aided procedures - and B (n = 80) - endoscope-aided surgery. No significant differences were reported between groups. However, the authors stressed that endoscope-aided resections resulted in minor clinically inactive residual lesions, thus reinforcing the quality of endoscope-aided cholesteatoma removal.

## DISCUSSION

In the opinion of the authors of this study, the distinction between recurrent and residual cholesteatoma is of fundamental importance to understand the mechanism by which relapsing disease is formed and controlled.

The site of recurrence may offer insights into the contributing factors for cholesteatoma formation.

The literature^20^ reports varying incidence rates of recurrent cholesteatoma, but the topography of involvement has been well established. The most commonly involved areas in decreasing order are the epitympanic recess, the tympanic cavity, the mastoid antrum, and the mastoid^20^.

Recurrent cholesteatomas pose threats only when tympanoplasty is performed to manage initial disease. Many authors have reported incidence rates under five percent when the following measures were considered: prevent the formation of adhesions between the medial surface of the graft and the bare bone surface of the epitympanum, mesotympanum, and facial recess; repair continuity defects of the bone portion of the ear canal and ensure good aeration of the middle ear, particularly in cases of eustachian tube disorder^10^.

Sheehy et al.^10^ described a procedure in which a large opening on the facial recess is made to pass a small plastic plate through the posterior tympanotomy to coat the exposed bone tissue and prevent graft adhesion. Bone defects of the outer ear canal and epitympanic recess can be repaired with chips of tragal cartilage. Reported incidence rates dropped from 21% to five percent after these measures were taken. Yanagihara et al.^5^ described good outcomes with the use of artificial dura mater fixated with fibrin glue to repair large scutum bone defects.

Despite its relatively low incidence rate and the declining rates of recurrent cholesteatoma, residual cholesteatomas of the middle ear are strongly correlated with recurrent disease. In reoperated patients, Shelton and Sheehy^21^ found residual cholesteatoma in one third of the subjects offered microscope-aided surgery.

Sheehy et al.^10^ looked into 303 revision surgery patients and identified relapsing disease in 36% of the subjects submitted originally to CWU mastoidectomy. The authors also reported higher incidence rates in children and confirmed the middle ear as the preferred site of involvement, followed by the epitympanum and, less commonly, the mastoid.

Smyth^22^ described an incidence of 13% of recurrent cholesteatomas in the epitympanum in 97 cases in which cholesteatomas were believed to have been completely removed. Residual cholesteatoma was seen in 12% of 208 examined middle ears. The late postoperative examination of children unsuspected for recurrent disease revealed an incidence of 23% of residual cholesteatoma.

Why are the incidence rates of postoperative residual cholesteatoma so high, and why are they found more commonly in the crevices of the middle ear and the epitympanic recess?

Initially, the lack of consensus on the choice between CWU or CWD mastoidectomy lied in the center of discussions on the management of cholesteatomas^23^. CWD mastoidectomy was claimed to result in a cavity conducive to otorrhea and constant postoperative care. By its turn, CWU mastoidectomy, still in development at that time, seemed to respect the anatomy of the middle ear at the expense of increased incidences of residual and recurrent cholesteatoma^19^.

Complete cholesteatoma removal is one of the main goals in the management of chronic middle ear processes. Ear microsurgery has developed significantly since the introduction of surgical microscopes. Two of the main features of surgical microscopes are parallel optical route and linear optical axis. However, the middle ear and the mastoid are embedded in the temporal bone and form a complex tridimensional structure, with a number of barely accessible anatomic landmarks. The poor lighting and limited viewing of the site of surgery provided by surgical microscopes prevent lesions located in deep lateral recesses of the middle ear from being easily removed, possibly leading to increased rates of residual cholesteatoma^12^. Conventional surgical approaches, given the limits of microscope-aided procedures, often require the drilling of uninvolved areas to allow access to specific sinuses or recesses, thus harming the principle of conservatism in temporal bone surgery^24^.

Residual disease tends to develop in sites where access to cholesteatoma is impeded in primary surgery^13^. Certain regions of the middle ear such as the anterior epitympanum and the supratubal recess, even after extensive mastoidectomy and exteriorization of the attic and the antrum, are difficult to access^19^. Despite the inherent risk of facial nerve injury, posterior tympanotomy provides access to the oval window and the facial recess, but fails to allow proper visualization of the deeper tympanic sinus during surgery^9^.

Donaldson et al.^25^ observed that the tympanic sinus, located medially to the facial nerve, may extend to well beyond the posterior margin of the fallopian canal. Thus, cleaning this anatomic site with any known instrument could be an extremely difficult task^26^. The longitudinal axis of the tympanic sinus runs perpendicularly to the ear canal, rendering visualization with a microscope impossible. According to Thomassin^9^, only by using a 70-degree endoscope through the transcanal approach can surgeons attain god visualization of the tympanic sinus.

The regions difficult to access in CWU mastoidectomy, particularly when the transmeatal approach is chosen, are the preferred sites for residual cholesteatoma.

The endoscopic transtympanic approach to the middle ear was originally described by Nomura^27^. Thomassin et al.^9^ described endoscopy-guided ear surgery as a complementary technique to mastoid surgery.

Endoscopic visualization usually covers the entire tympanic ring and the ear canal in the same field of vision. Although microscopes offer tridimensional visualization, their field of vision is limited by the narrower portion of the ear canal. This hampers visualization and leads to constant manipulation of the microscope or the patient's head during surgery^14^.

Endoscopy is an excellent addition to the toolset used to assess the middle ear, particularly when areas difficult to view using a microscope such as the tympanic sinus, facial recess, hypotympanum, epitympanum, and supratubal recess are considered^14^, ^15^. The removal of residual cholesteatomas in these sites is possible when specific ear endoscopy instruments are used^9^, ^26^.

The steps described for endoscope-assisted surgery are very similar among most authors. After the completion of traditional microscope-aided procedures, inspection with an endoscope is made in anatomic sites of difficult access. Using a 3-mm, 16-cm, 45° endoscope and the transcanal approach, Marchioni^26^ described excellent control of the medial border of the tympanic sinus, oval window niche, junction of the styloid eminence and the jugular bulb, and the entire inferior retrotympanum and hypotympanum. Thomassin et al.^9^ described total control of the tympanic cavity with 2.7 mm, 0° and 70° endoscopes using the transcanal or transmastoid approaches.

However, some shortcomings of the endoscopic technique may limit its use in ear surgery. The lack of tridimensional visualization requires surgeons to be particularly careful when handling surgical equipment. Additionally, the surgeon has only one free hand and bleeding may render the procedure unviable^14^.

The use of ear endoscopy as an ancillary method during surgery improved the assessment of the cavity after the complete removal of the cholesteatoma with the aid of a microscope. Ayache et al.^13^ found evidences of residual tissue in the epitympanum and retrotympanum in 44% and 76% of the cases respectively. El-Meselaty et al.^18^ reported residual cholesteatoma in 50% of the patients submitted to CWU mastoidectomy. Badr-el-Dine^16^ reported an incidence rate of 23.8% in a series of 82 cases of CWU mastoidectomy.

The contributions of intraoperative ear endoscopy in cholesteatoma surgery are evident. However, in practical terms, has endoscopy actually reduced the incidence of recurrent disease even in more conservative approaches?

Many authors have observed significant reductions on both incidence rates and extent of lesion when endoscopes were used. Yung^28^, M. Badr-el-Dine^16^, and Barakate & Bottrill^17^ reported incidence rates of residual cholesteatoma of 9.4%, 8.6%, and 15.78% respectively in revision surgery after CWU mastoidectomy.

Thomassin et al.^9^ compared endoscope-aided to traditional ear microsurgery and found reductions on the rates of residual cholesteatoma from 47% to 6% in a group offered intraoperative endoscopy followed up for 19 months. A “third-look” procedure was required in 10% of the patients offered traditional surgery because of the size of their cholesteatomas. El-Meselaty et al^18^ did not report recurrence in patients operated with the aid of an endoscope, against recurrence rates of 26% and 5% in patients offered CWU and CWD mastoidectomy respectively.

Ayache et al.^19^ did not find significant differences between patients submitted to microscope-aided surgery and subjects offered endoscope-aided procedures. However, the use of the endoscope produced clinically inactive pearl-shaped residual lesions, thus stressing the good quality of disease removal when ear endoscopy was used as an ancillary method.

The significant variability in the incidence of residual cholesteatoma may be explained by factors connected to surgeon experience and skill level, the time until revision surgery for residual cholesteatoma, or even the specific indication criteria each author used to choose CWU instead of CWD mastoidectomy. Another interesting aspect is that late revision surgery possibly yields higher and certainly more realistic recurrence incidence rates.

## CONCLUSION

None of the papers considered in this study were randomized trials. Nonetheless, they described several advantages of using ear endoscopy in the management of cholesteatomatous chronic otitis media. Even when recurrence rates were not decreased, authors reported that recurrent cholesteatomas were more easily located and surgically managed in endoscope-aided procedures. Therefore, it appears that endoscopy should be incorporated in the daily practice of otology.
